# Assessing mortality risk in colorectal cancer survivors with congestive heart failure: a cohort study from the Taiwan Cancer Registry

**DOI:** 10.1186/s12885-026-16717-5

**Published:** 2026-08-12

**Authors:** Mitsuhiro Koseki, Ching-Chieh Yang, I-Che Feng, Wei-Chieh Lee, Chien‑Liang Lin, Ying-Jia Lin, Jhi-Joung Wang, Chung-Han Ho

**Affiliations:** 1 Division of Gastroenterology and Hepatology, Department of Internal Medicine, Chi Mei Medical Center, Tainan, Taiwan; 2 Department of Internal Medicine, Chi Mei Medical Center, Tainan, Taiwan; 3 Department of Radiation Oncology, Chi Mei Medical Center, Tainan, Taiwan; 4 Department of Pharmacy, Chia-Nan University of Pharmacy and Science, Tainan, Taiwan; 5 Cancer Center, Chi Mei Medical Center, Tainan, Taiwan; 6 Division of Cardiology, Department of Internal Medicine, Chi Mei Medical Center, Tainan, Taiwan; 7 Division of Hematology‑Oncology, Department of Internal Medicine, Chi Mei Hospital, Liouying District, Tainan, Taiwan; 8 Department of Medical Research, Chi Mei Medical Center Tainan, Tainan City, Taiwan; 9 Department of Information Management, Southern Taiwan University of Science and Technology, Tainan, Taiwan; 10 Cancer Center, Taipei Municipal Wanfang Hospital, Taipei Medical University, Taipei, Taiwan

**Keywords:** Colorectal cancer, Mortality, Chronic heart failure, Real-world database, Taiwan Cancer Registry

## Abstract

**Background:**

The rising incidence of colorectal cancer (CRC) and cardiovascular diseases, driven by aging populations and lifestyle changes, highlights congestive heart failure (CHF) as a critical comorbidity that significantly affects survival and treatment decisions in CRC patients. CHF not only affects survival outcomes but also complicates clinical decision-making. This study aims to investigate the impact of CHF on mortality among CRC survivors and explore potential factors of mortality.

**Methods:**

This cohort study analyzed data from the Taiwan Cancer Registry and National Health Insurance Research Database. CRC patients aged 20 years or older (ICD-10-CM: C18–C20) who survived at least five years after diagnosis were included. After a 1:4 exact matching based on age, sex, and clinical stage, Cox regression was used to estimate mortality risk between CRC survivors with and without CHF.

**Results:**

A total of 535 CRC survivors with CHF and 2140 without CHF were enrolled in this study after matching. CRC survivors with CHF had a significantly higher overall mortality rate than those without CHF (36.64% vs. 15.23%, *p* < 0.001). After adjusting for potential confounders, CRC survivors with CHF had 1.93-folds risk of mortality (95% CI: 1.54–2.41, *p* < 0.001) compared to those without CHF. Subgroup analyses by sex, age, clinical stage, Charlson Comorbidity Index (CCI), and treatment types also show that CRC survivors with CHF had significantly higher mortality risk than those without CHF. The top three highest mortality risk ratios were observed in subgroups with a CCI score of 0 (AHR: 2.63, 95% CI: 1.08–6.39), age < 65 years (AHR: 1.87, 95% CI: 1.23–5.57), and advanced clinical stage (AHR: 2.03, 95% CI: 1.51–2.72).

**Conclusion:**

CRC survivors with CHF demonstrated significantly higher overall mortality compared to those without CHF. This finding presented the prognostic impact of CHF in CRC survivors, so integrated care and early cardiovascular monitoring are essential to improve long-term outcomes.

## Introduction

The global trends of increasing population aging and the adoption of Westernized lifestyles have contributed to a rising incidence of colorectal cancer (CRC) and cardiovascular diseases (CVDs) [1, 2]. As a result, cardiovascular risk factors, such as hypertension, hyperglycemia, hyperlipidemia, and obesity, have become increasingly among newly diagnosed CRC patients [3]. This is particularly relevant because congestive heart failure (CHF), a common comorbidity in CRC, is a major contributor to overall mortality [4, 5]. Older adults, who are commonly affected by CHF, also accepted a higher burden of CRC [6, 7].

Investigating the association between CHF and CRC is imperative, as the coexistence of these comorbid conditions profoundly influences treatment decisions and patient outcomes. Especially, patients with both cancer and CHF often face significantly worse prognoses due to the combined burden of disease. A population-based cohort study from Taiwan reported a significantly higher incidence of CVDs in CRC patients, particularly within the first year after diagnosis [8]. This finding highlights the importance of early cardiovascular monitoring in CRC patients, as the risk of coronary heart disease remains elevated, while the risk of ischemic stroke appears to decline after the second year.

Given the significant comorbidity of CHF among CRC patients, integrated health management strategies are urgently needed to address the overlapping risks of cardiovascular and oncological. A primary care-based cohort study highlighted the poor prognosis associated with CHF, demonstrating worse survival outcomes than those of breast and prostate cancer, but better or comparable outcomes relative to lung and colorectal cancer [9]. This underscores the substantial comorbidity burden in CHF patients, which critically affects survival and necessitates tailored, multidisciplinary management strategies.

Therefore, this study aims to investigate the impact of CHF on mortality in long-term CRC survivors and to identify potential factors associated with mortality risk. Understanding this relationship is crucial, as CHF may significantly affect both treatment decisions and survival outcomes in CRC survivors. The findings could further emphasize the importance of integrating cardiovascular and oncological management to optimize care and improve outcomes among CRC survivors.

## Material and methods

### Data source

In this study, data from the TCR and the NHIRD were analyzed. The TCR, established in 1979, is well-regarded for its high-quality, accurate records on cancer diagnoses and treatments, making it a key resource for tracking cancer incidence and mortality trends in Taiwan. In 2002, the TCR was expanded to include detailed datasets on cancers of the colon, rectum, oral cavity, pharynx (excluding nasopharynx), liver, lung, breast, and cervix. In 2007, prostate cancer data were also added to the registry [10, 11]. Data on comorbidities and medical histories were obtained from the NHIRD, which encompasses all inpatient and outpatient healthcare services under Taiwan's National Health Insurance program. To support research initiatives, the Health and Welfare Data Science Center in Taiwan integrated this population database with multiple health-related datasets. This process was carefully managed to ensure full compliance with personal data protection regulations [12].

### Ethical statements

In this study, all participant data were rendered anonymous to maintain confidentiality. The study was conducted in full accordance with the Declaration of Helsinki and received approval from the Institutional Review Board (IRB) of Chi Mei Medical Center (IRB serial no. 11206–002). The requirement for patient informed consent was waived by the IRB of CMMC due to the study's retrospective and observational design, with assurance that patient welfare would not be compromised.

### Study population

This study used a real-world population-based cohort design to select colorectal cancer patients aged 20 years or older. The diagnosis of colorectal cancer was based on the ICD-10-CM (International Classification of Diseases, Tenth Revision, Clinical Modification) codes, C18-C20. These patients, who had survived more than five years following their initially diagnosis, were identified from the TCR between 2017 and 2019. The index date of these patients was the date of their fifth year after the initially diagnosis date of colon cancer. For reducing the misclassification bias, the selected variables with missing values were all excluded to ensure data integrity.

**Fig. 1:**
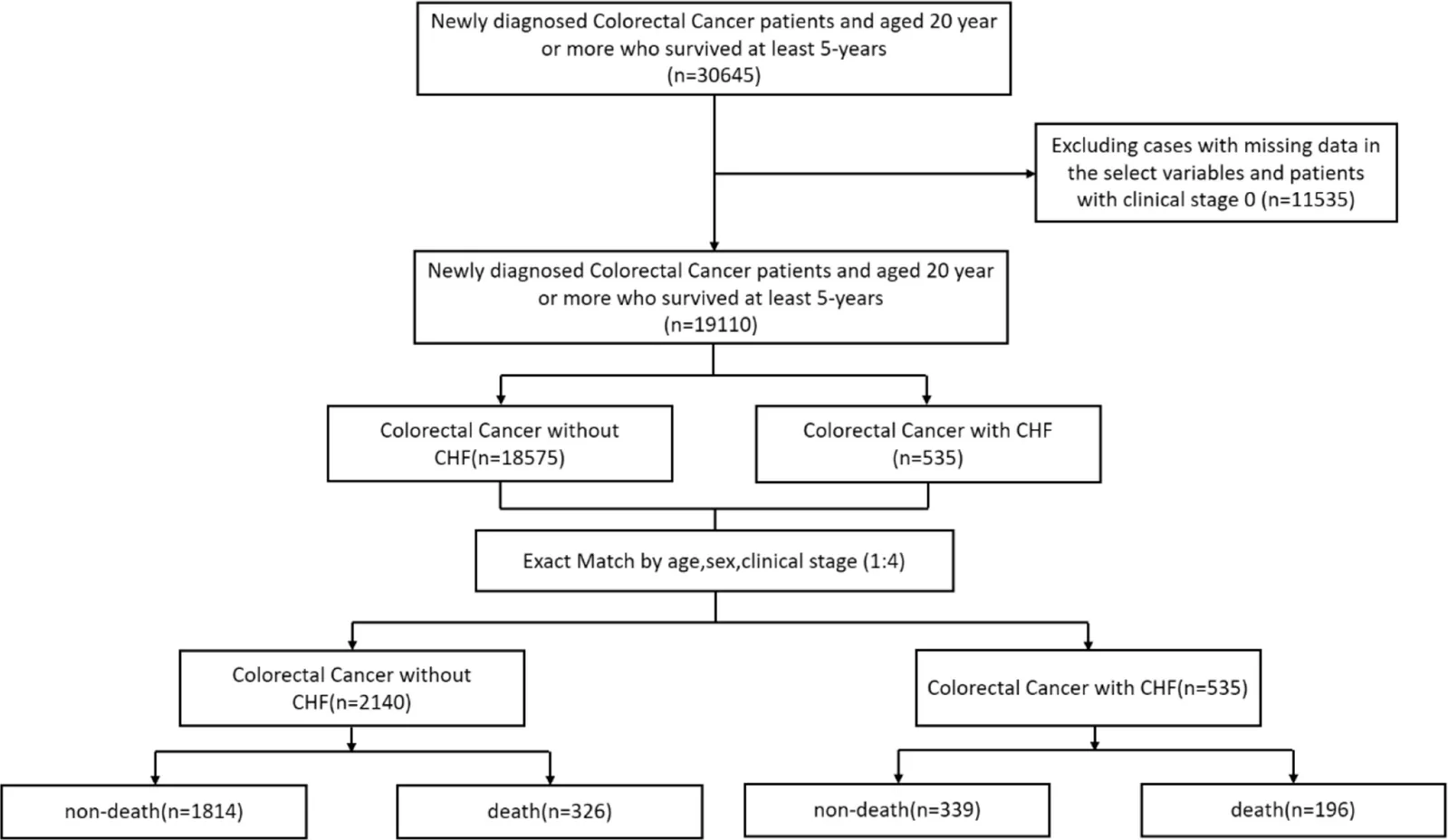
Flow chart of study subjects selection

Based on the study objectives, the CRC survivors were categorized into two groups: those with a history of CHF and those without. CHF was defined according to hospital records from the year before the colorectal cancer diagnosis, using ICD-10-CM codes I09.9, I11.0, I13.0, I13.2, I25.5, I42.0, I42.5-I42.9, I43, I50, and P29.0. To minimize potential confounding bias, the exact matching was applied to match the CHF cohort with comparison cohort, patients without CHF, at a 1:4 ratio under the factors including of age, sex, and cancer clinical stage. All subjects were all right-censored to the date of death or the end of follow-up date, December 31, 2021. The flowchart of study selection presented as Fig. 1.

### Outcome and measurements

The primary objective of this study was to assess the overall mortality among long-term CRC survivors between those with and without CHF. The mortality was identified using Taiwan's national cause-of-death database. To minimize the potential confounding bias, factors such as age, sex, body mass index (BMI), lifestyle factors (smoking and alcohol consumption), cancer clinical stage, treatment types (surgery, radiotherapy, chemotherapy), Charlson Comorbidity Index (CCI) score, myocardial infarction (MI), peripheral vascular disease (PVD), cerebrovascular accident (CVA), diabetes mellitus (DM), hyperlipidemia, and hypertension (HTN) were evaluated to estimate the mortality risk in study subjects.

Age was calculated at the index date, the fifth year after the initial CRC diagnosis. The CCI score was used to evaluate the burden of multimorbidity and serves as a crucial factor in assessing CRC survivors [13]. The CCI was calculated using ICD-10-CM diagnostic codes from the NHIRD during the 12-month period before the index date. Comorbidity was identified based on validated coding algorithms, and each was assigned a weight according to the original Charlson Comorbidity Index scoring system [14, 15]. The total CCI score was presented by summing the weights of all identified comorbid conditions. Therefore, in this study, the calculation of the CCI is based on patients diagnosed with colon cancer in their fourth year, who have attended three outpatient visits or been hospitalized once. CHF was excluded from the CCI score to avoid overlap with the main outcome of interest. Additionally, other comorbidities potentially associated with CHF, such as MI, PVD, CVA, DM, hyperlipidemia, and HTN, were also considered. These comorbidities were included if recorded during hospitalization or in at least three or more outpatient visits before the cancer diagnosis.

### Statistical analysis

All statistical analyses were performed using SAS 9.4 for Windows (SAS Institute, Inc., Cary, NC, USA) in this study. Continuous variables using independent sample t-tests and categorical variables using Pearson’s chi-square tests or Fisher’s exact tests were used to compare the baseline characteristics between CRC survivors with and without CHF.

Kaplan–Meier plots were used to examine the trend of mortality between long-term CRC survivors with and without CHF. Cox proportional hazards model with aggregated sandwich estimator was used to estimate hazard ratio (HR) and 95% confidence interval (CI) for the mortality risk after matching. Both crude and adjusted HRs were calculated. The adjusted models included covariates such as BMI, smoking, alcohol consumption, treatment modalities, CCI score, and comorbidities potentially associated with CHF. The results of the subgroup analyses were also illustrated using forest plots, stratified by age group, sex, cancer clinical stage, CCI score, and treatment type, providing a clear comparison across different patient characteristics. Additionally, to address potential confounding from other major cardiovascular comorbidities, a sensitivity analysis was presented by excluding CRC survivors with a history of MI or CVA before the index date. Kaplan–Meier curves were plotted using STATA version 12.0 (Stata Corp., College Station, TX, USA).

## Results

After matching, the final study population included 535 survivors in the CHF cohort and 2140 survivors in the comparison cohort without CHF. Table 1 show the baseline distribution between long-term CRC survivors with CHF and those without. The mean BMI remained significantly higher in the CHF group than comparison cohort without CHF (Mean: 25.17 ± 3.93 vs. 23.92 ± 3.92, *p* < 0.001), and a larger proportion of CHF patients had a BMI ≥ 25 group (47.66% vs. 36.40%, *p* < 0.001) compared with those without CHF. CHF patients continued to have higher rates of comorbidities, such as myocardial infarction (4.86% vs. 0.65%, *p* < 0.001), cerebrovascular accidents (14.21% vs. 7.85%, *p* < 0.001), diabetes (38.69% vs. 21.87%, *p* < 0.001), hyperlipidemia (26.54% vs. 17.62%, *p* < 0.001), and hypertension (74.58% vs. 37.29%, *p* < 0.001), than non-CHF group. Additionally, CHF patients had a higher rate of surgery treatment (96.64% vs. 92.57%, *p* = 0.001). Overall mortality was also significantly higher among CRC survivors with CHF compared to those without (36.64% vs. 15.23%; *p* < 0.001).

**Table 1:** Baseline characteristics of CRC patients between patients with CHF and those without

	Overall (*n* = 2675)	Non-CHF (*n* = 2140)	CHF (*n* = 535)	*p*-value*
Age, Mean ± SD	75.73 ± 11.17	75.66 ± 11.20	76.01 ± 11.09	0.519
Age group				1.000
< 50	55(2.06)	44(2.06)	11(2.06)	
50–59	140(5.23)	112(5.23)	28(5.23)	
60–69	580(21.68)	464(21.68)	116(21.68)	
70–79	765(28.60)	612(28.60)	153(28.60)	
≥ 80	1135(42.43)	908(42.43)	227(42.43)	
Sex				1.000
Male	1585(59.25)	1268(59.25)	317(59.25)	
Female	1090(40.75)	872(40.75)	218(40.75)	
BMI, Mean ± SD	24.17 ± 3.95	23.92 ± 3.92	25.17 ± 3.93	< 0.001
BMI group				< 0.001
< 18.5	181(6.77)	165(7.71)	16(2.99)	
18.5–24	1460(54.58)	1196(55.89)	264(49.35)	
≥ 25	1034(38.65)	779(36.40)	255(47.66)	
Smoking				0.416
Never	1972(73.72)	1585(74.07)	387(72.34)	
Ever/current	703(26.28)	555(25.93)	148(27.66)	
Alcohol consumption				0.829
Never	2129(79.59)	1705(79.67)	424(79.25)	
Ever/current	546(20.41)	435(20.33)	111(20.75)	
Cancer clinical stage				1.000
Stage I	905(33.83)	724(33.83)	181(33.83)	
Stage II	645(24.11)	516(24.11)	129(24.11)	
Stage III	1050(39.25)	840(39.25)	210(39.25)	
Stage IV	75(2.80)	60(2.80)	15(2.80)	
CCI**				< 0.001
CCI = 0	712(26.62)	675(31.54)	37(6.92)	
CCI = 1,2	831(31.07)	666(31.12)	165(30.84)	
CCI≧3	1132(42.32)	799(37.34)	333(62.24)	
Treatment				
Surgery	2498(93.38)	1981(92.57)	517(96.64)	0.001
Radiotherapy	318(11.89)	266(12.43)	52(9.72)	0.083
Chemotherapy	1374(51.36)	1106(51.68)	268(50.09)	0.511
Comorbidity				
MI	40(1.50)	14(0.65)	26(4.86)	< 0.001
PVD	48(1.79)	31(1.45)	17(3.18)	0.007
CVA	244(9.12)	168(7.85)	76(14.21)	< 0.001
DM	675(25.23)	468(21.87)	207(38.69)	< 0.001
Hyperlipidemia	519(19.40)	377(17.62)	142(26.54)	< 0.001
HTN	1197(44.75)	798(37.29)	399(74.58)	< 0.001
Death	522(19.51)	326(15.23)	196(36.64)	< 0.001
Time to death (year), Mean ± SD	1.67 ± 1.15	1.73 ± 1.19	1.56 ± 1.08	0.090

^*^Continuous variables were analyzed using the independent samples t-test, and categorical variables were analyzed using the Pearson’s chi-square test or Fisher’s exact test

^**^ CHF was not included in the CCI

**Fig. 2:**
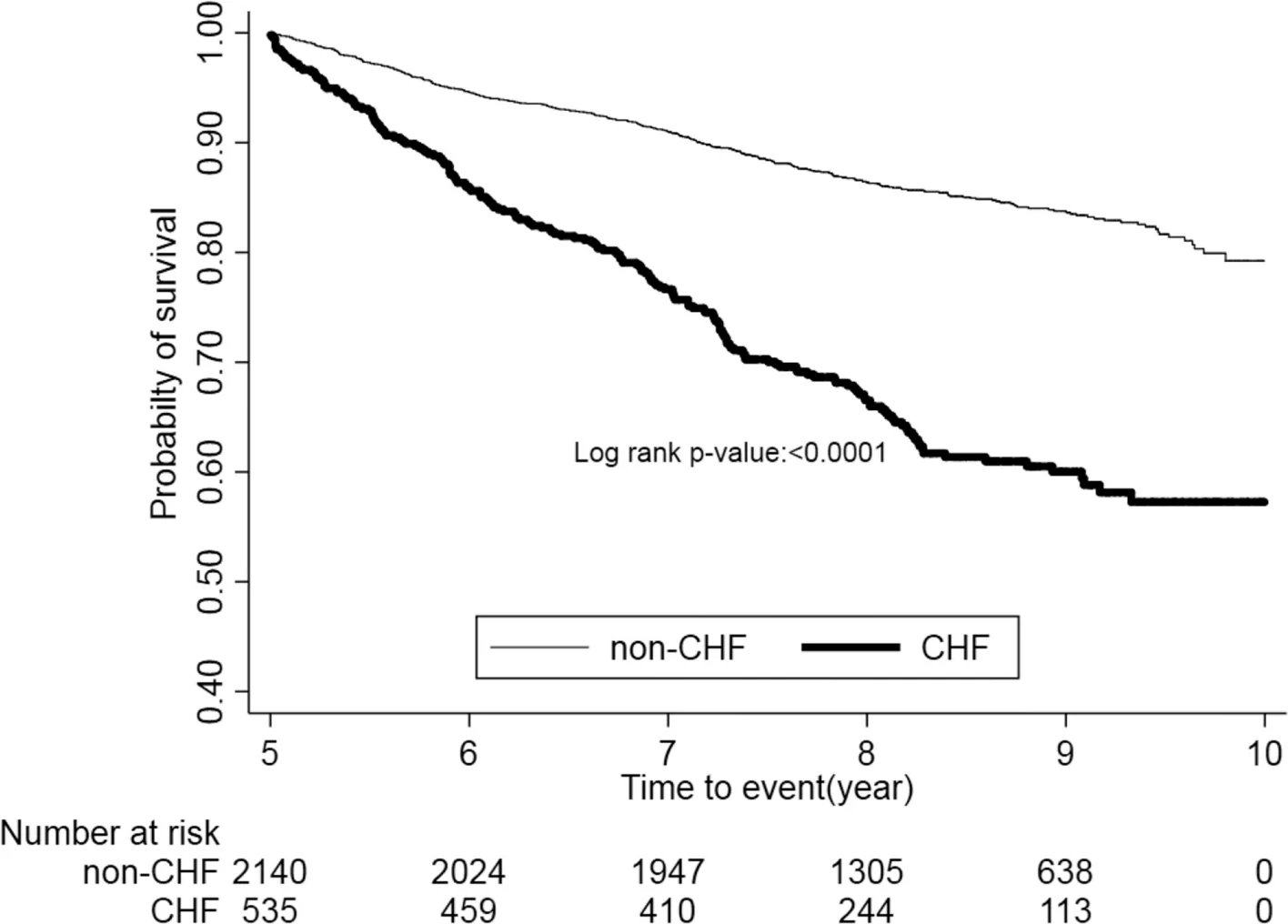
Kaplan–Meier plot of overall survival for CRC patients with and without CHF

The Kaplan–Meier survival curve illustrates the differences in overall survival probability for CRC survivors with CHF compared to those without CHF after index date (Fig. 2). The log-rank test demonstrates a statistically significant difference in survival between these two groups (*p* < 0.001). CRC survivors without CHF exhibited consistently higher survival outcomes, with a higher probability of survival throughout the follow-up period. The survival probability declined more steeply in the CHF group, indicating worse outcomes associated with CHF in CRC survivors.

**Fig. 3:**
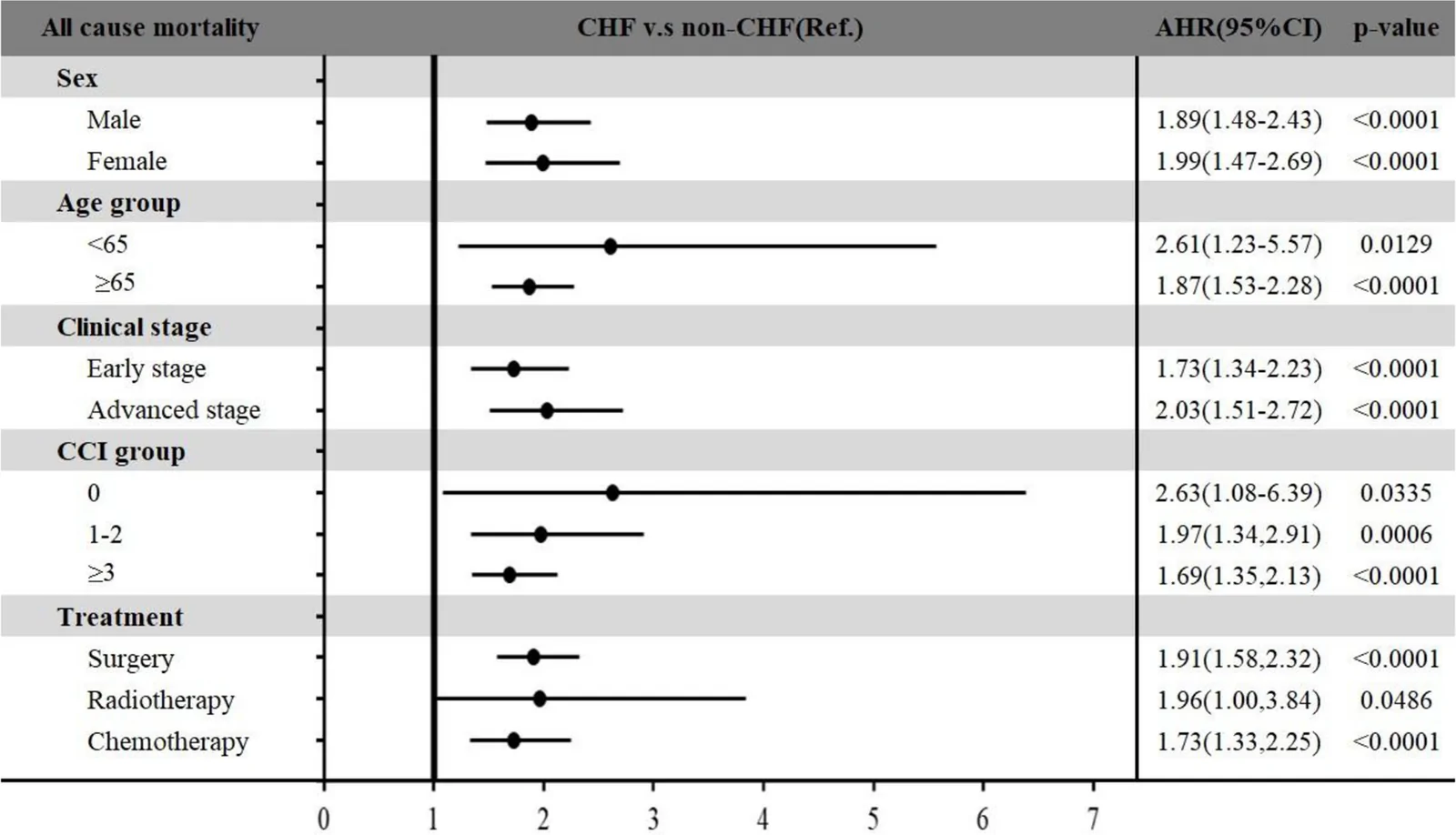
Forest plot of subgroup analysis for overall survival

Table 2 indicated that the risk of overall survival between CRC survivors with and without CHF. After the adjustment with selected confounding factors, CRC survivors with CHF had a 1.93-fold significantly higher mortality risk compared to those without CHF (adjusted HR = 1.93, 95% CI: 1.54–2.41, *p* < 0.001). The forest plot illustrates the adjusted HR for various subgroups, including of sex, age, clinical stage, CCI, and treatment types, influencing all-cause mortality in CRC survivors (Fig. 3). Among CRC survivors with CHF, the top three subgroups with the highest mortality risk were those with a CCI score of 0 (AHR: 2.63, 95% CI: 1.08–6.39, *p* = 0.0335), age < 65 (AHR: 1.87, 95% CI: 1.23–5.57, *p* = 0.0129), and advanced clinical stage (AHR: 2.03, 95% CI: 1.51–2.72, *p* < 0.001), compared to the non-CHF cohort.

**Table 2:** The risk of overall survival between CRC patients with and without CHF

	Crude HR	*p*-value	Adjusted HR	*p*-value
CHF				
No	ref		ref	
Yes	3.08(2.53,3.74)	< 0.001	1.93(1.54,2.41)	< 0.001
BMI group				
< 18.5	0.96(0.64,1.43)	0.836	1.62(1.03,2.55)	0.036
18.5–25	ref		ref	
≥ 25	1.18(0.95,1.46)	0.144	1.00(0.78,1.28)	0.978
Smoking				
Never	ref		ref	
Ever/current	1.19(0.92,1.53)	0.184	1.52(1.11,2.07)	0.009
Alcohol consumption				
Never	ref		ref	
Ever/current	0.97(0.74,1.27)	0.823	0.76(0.55,1.05)	0.090
Treatment				
Surgery	3.05(1.80,5.17)	< 0.001	1.56(0.86,2.82)	0.143
Radiotherapy	0.93(0.67,1.29)	0.659	1.04(0.71,1.53)	0.833
Chemotherapy	1.39(1.12,1.74)	0.003	1.23(0.95,1.58)	0.113
CCI group*				
CCI = 0	ref		ref	
CCI = 1,2	4.16(2.85,6.07)	< 0.001	3.34(2.23,5.01)	< 0.001
CCI ≥ 3	8.46(5.98,11.98)	< 0.001	6.56(4.33,9.95)	< 0.001
Comorbidities				
MI	4.01(1.95,8.25)	0.002	1.75(0.78,3.92)	0.177
PVD	1.59(0.82,3.12)	0.173	0.86(0.41,1.79)	0.684
CVA	1.78(1.31,2.42)	0.002	0.85(0.60,1.20)	0.351
DM	2.10(1.69,2.61)	< 0.001	0.85(0.64,1.12)	0.242
Hyperlipidemia	1.25(0.98,1.60)	0.075	0.81(0.61,1.08)	0.159
HTN	2.71(2.20,3.34)	< 0.001	1.37(1.06,1.76)	0.016

^*^CHF was not included in the CCI

Table 3 show the sensitivity analyses of excluding CRC survivors with a history of MI or CVA before the index date. The results remained consistent with the main findings for overall and subgroup analysis. CRC survivors with CHF exhibited a significantly 2.02-folds risk of all-cause mortality compared to those without CHF (95% C.I.: 1.55–2.63, *p* < 0.0001). Subgroup analysis show that CRC survivors with CHF aged < 65 (AHR: 2.89, 95% CI: 1.27–6.58, *p* = 0.0113) and those with a CCI score of 0 (AHR: 2.71, 95% CI: 1.12–6.59, *p* = 0.0275) had the two highest mortality risks compared to non-CHF patients.

**Table 3:** Sensitivity analyses of overall mortality excluding CRC survivors with a history MI or CVA

	Non-CHF	CHF	AHR(95%CI)	*p*-value
Overall	1961	439	2.02(1.55–2.63)	< 0.001
Stratified analysis				
Sex				
Male	1150	247	1.78(1.35–2.35)	< 0.001
Female	811	192	1.91(1.37–2.64)	< 0.001
Age group				
Age < 65	343	71	2.89(1.27–6.58)	0.011
Age > = 65	1618	368	1.78(1.43–2.22)	< 0.001
Clinical stage				
Early stage	1132	255	1.64(1.23–2.19)	< 0.001
Advanced stage	829	184	2.00(1.46–2.74)	< 0.001
CCI group				
0	675	37	2.71(1.12–6.59)	0.028
1–2	637	150	2.06(1.37–3.10)	< 0.001
≥ 3	649	252	1.68(1.31–2.17)	< 0.001
Treatment				
Surgery	1807	426	1.87(1.52–2.31)	< 0.001
Radiotherapy	254	45	2.05(0.99–4.26)	0.054
Chemotherapy	1019	225	1.59(1.20–2.12)	0.001

^*^Adjusted BMI, smoking, Alcohol consumption, treatment, CCI, PVD, DM, Hyperlipidemia, HTN

## Discussion

In our study, CRC survivors with CHF show a significantly higher overall mortality than those without CHF (36.64% vs. 15.23%, *p* < 0.001). After adjusting for key confounders, CRC survivors with CHF had a 1.93-fold higher risk of mortality compared to the non-CHF cohorts (AHR = 1.93, 95% CI: 1.54–2.41, *p* < 0.001). These results indicated the impact of CHF on mortality among CRC survivors, and addressing CHF was a critical factor in improving survival outcomes, even after considering critical demographic and clinical factors.

Previous studies have established that CHF alone confers a substantial mortality burden, with 5-year survival rates typically ranging from 50–60% and estimated 10-year survival rates below 30% in community-based cohorts [9, 16]. However, much of the existing literature focuses on CHF in populations without cancer. Our findings demonstrated that among colorectal cancer survivors, comorbid CHF significantly worsens overall survival, even after adjusting for age, sex, and cancer stage. These findings highlight the compounded prognostic impact of CHF when superimposed on cancer survivorship.

The study specifically focused on long-term CRC survivors, defined as those who survived at least five years following diagnosis. This inclusion criterion was chosen to examine the impact of CHF beyond the acute treatment phase, which is often complicated by treatment-related adverse effects and cancer-related mortality. By excluding early deaths, our design aimed to estimate the chronic impact of CHF on long-term survival in a more stable survivorship population. Although this may introduce survivorship bias, it could help us to understand how CHF could affect the late mortality risks in cancer survivors, a group that is growing rapidly due to advances in cancer treatment and aging populations.

The cardiovascular burden among CRC patients is worsened by cancer treatments interacting with existing cardiovascular risk factors [17]. Chemotherapy has been associated with an increased risk of heart failure, especially in elderly patients with diabetes or hypertension [18]. A multi-registry cohort study found a significant incidence of major adverse cardiovascular events (MACCE) among CRC patients undergoing surgery, especially for those with pre-existing diabetes and cardiovascular disease, and chemotherapy was paradoxically associated with a reduced long-term MACCE risk [19]. Furthermore, a nationwide cohort study of CRC patients receiving fluoropyrimidine-based adjuvant chemotherapy, the tegafur-uracil group had higher rates of heart failure and ischemic stroke, especially in patients aged 70 and older with stage III disease [20]. These findings indicated the need for proactive cardiovascular monitoring in CRC patients, particularly those receiving systemic therapy.

Consistent with our findings, other studies have also indicated the overall poor prognosis associated with CHF, which may decrease survival rates in CRC patients with this comorbidity [9, 21]. The progression of heart failure may further limit survival because comorbidities like CHF significantly impact overall survival in CRC patients, even after treatment [22]. Emerging evidence in cardio-oncology also suggested that heart failure phenotype significantly had affected outcomes in cancer patients, with HFpEF generally associated with better prognosis than HFrEF [23, 24]. Fibrosis, oxidative stress, and inflammation are key mechanisms shared by CRC progression and CHF, potentially explaining the higher mortality observed in CRC survivors with CHF. In CRC, tumor progression is facilitated by transforming growth factor-beta (TGF-β)-driven fibrosis and inflammatory cytokines, while in CHF, fibrotic cardiac remodeling impairs myocardial function [25]. Similarly, oxidative stress contributes to DNA damage and inflammation in CRC and worsens myocardial injury in CHF [26]. Elevated circulating cytokines such as interleukin-6 (IL-6) and tumor necrosis factor-alpha (TNF-α) may link systemic inflammation in CHF to cancer progression [27]. Our findings show the impact of these mechanisms on survival outcomes, emphasizing the need for integrative therapeutic strategies targeting fibrosis, oxidative stress, and inflammation to improve the prognosis of CRC patients with CHF.

After using the real-word longitudinal population database, this study offers powerful evidence on the mortality risk among CRC survivors with CHF. However, this study has several limitations. First, the potential survivorship bias of this cohort study should be considered. As we focus on long-term CRC survivors, the enrolled study population may be healthier CRC patients, potentially limiting the generalizability of our findings to those with more severe comorbidities or advanced disease. To reduce this bias, the comorbidity burden using the CCI score was adjusted to estimate the mortality risk in this study; however, residual confounding may still exist. Second, the study did not examine CHF subtypes due to the lack of echocardiographic data, such as left ventricular ejection fraction (LVEF). LVEF rates played an important role to affect the outcome of survival [28].Consequently, we were unable to divide between heart failure with reduced ejection fraction (HFrEF) and preserved ejection fraction (HFpEF). Previous studies have indicated that HFrEF in cancer survivors is linked to worse outcomes, including increased hospitalization and mortality, compared to HFpEF [23, 24]. This limitation may affect interpretation of the heterogeneity in CHF impact on mortality among CRC survivors. Third, while the study adjusted for comorbidities using CCI score, it did not explore the individual impact of specific conditions, such as DM or HTN, which may independently affect survival outcomes [29, 30]. Similarly, although this study included the variables of smoking and alcohol consumption, the database did not have enough information on lifestyle factors and socioeconomic status, which are known to affect long-term mortality. Future research should address these gaps by incorporating more detailed clinical and lifestyle data, expanding subgroup analyses, and investigating the impact of specific comorbidities and treatment regimens. Fourth, the study assessed only all-cause mortality. This limits the ability to distinguish between deaths attributable to cardiovascular causes, cancer recurrence, or other conditions, and may hide the specific contribution of CHF to mortality risk. In the future research, we should add the information of cause-specific mortality for better estimation. Lastly, potential mechanisms such as inflammation, oxidative stress, and fibrosis may link CHF and cancer progression, and certain CHF medications may contribute to these effects [31–34]. These interpretations remain speculative and are not directly supported by our data. Future studies with detailed medication and biological data are needed to validate these associations. That will improve the understanding of survival outcomes in CRC survivors with CHF and guide more personalized management strategies.

## Conclusion

In conclusion, CRC survivors with CHF demonstrated significantly higher overall mortality compared to those without CHF. The high mortality rates presented the significant impact of CHF on long-term survival in CRC survivors. These findings suggest that cardiovascular comorbidities, especially CHF, exacerbate mortality risks, potentially due to the interactions between cancer therapies and underlying cardiovascular disease. The complexity of managing CRC patients with CHF presented the need for routine cardiovascular monitoring and integrated, multidisciplinary care strategies. Future efforts should focus on improve management approaches to address the compounded risks for this population.

## Data Availability

The data that support the findings of this study are available from the Collaboration Center of Health Information Application, but restrictions apply to the availability of these data, which were used under license for the current study, and so are not publicly available. Data are however available from the authors upon reasonable request and with permission of the Ministry of Health and Welfare.

## Abbreviations

CRC: Colorectal Cancer
CHF: Congestive heart failure
TCR: Taiwan Cancer Registry
NHIRD: National Health Insurance Research Database
HR: Hazard ratio
CI: Confidence intervals
CCI: Charlson Comorbidity Index
CVDs: Cardiovascular diseases
SIR: Standardized incidence ratio
IRB: Institutional Review Board
CMMC: Chi Mei Medical Center
ICD-10-CM: International Classification of Diseases, Tenth Revision, Clinical Modification
BMI: Body Mass Index
OR: Odds ratios
CI: Confidence interval
MI: Myocardial infarction
PVD: Peripheral vascular disease
CVA: Cerebrovascular accident
DM: Diabetes mellitus
HTN: Hypertension
MACCE: Major adverse cardiovascular events
LVEF: Left ventricular ejection fraction
